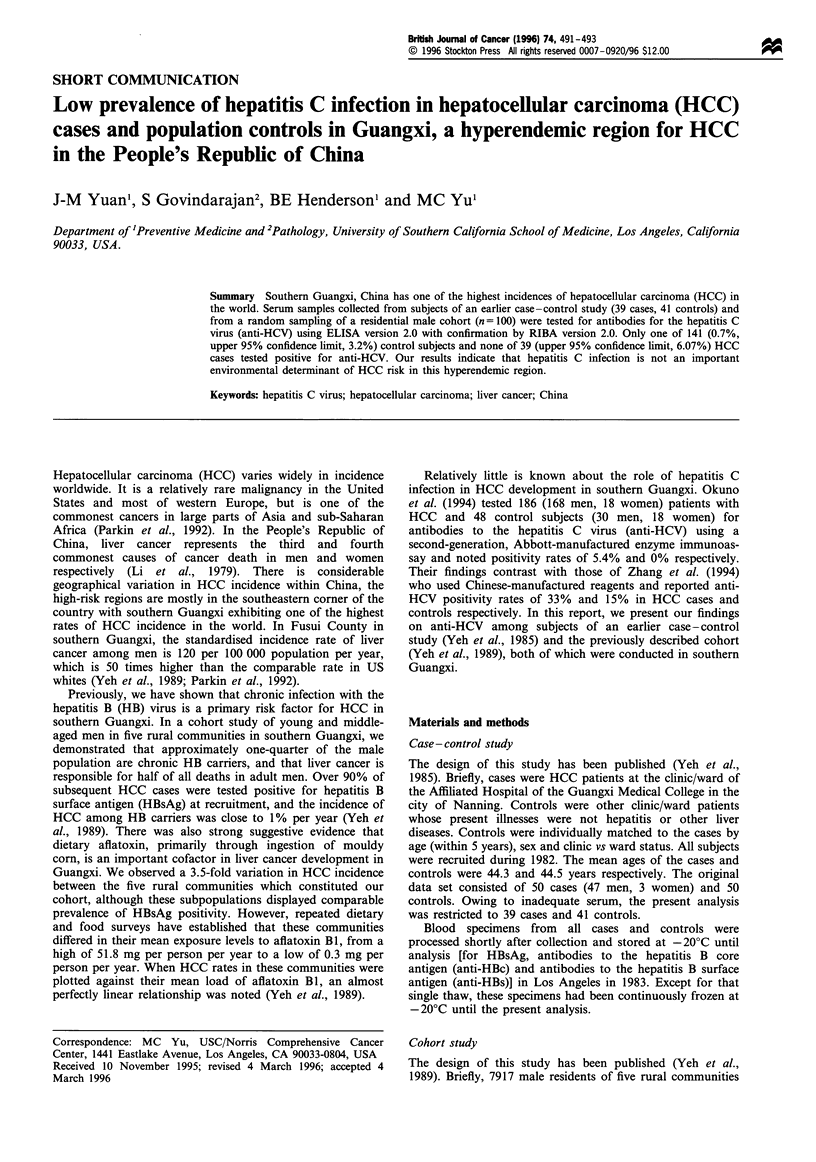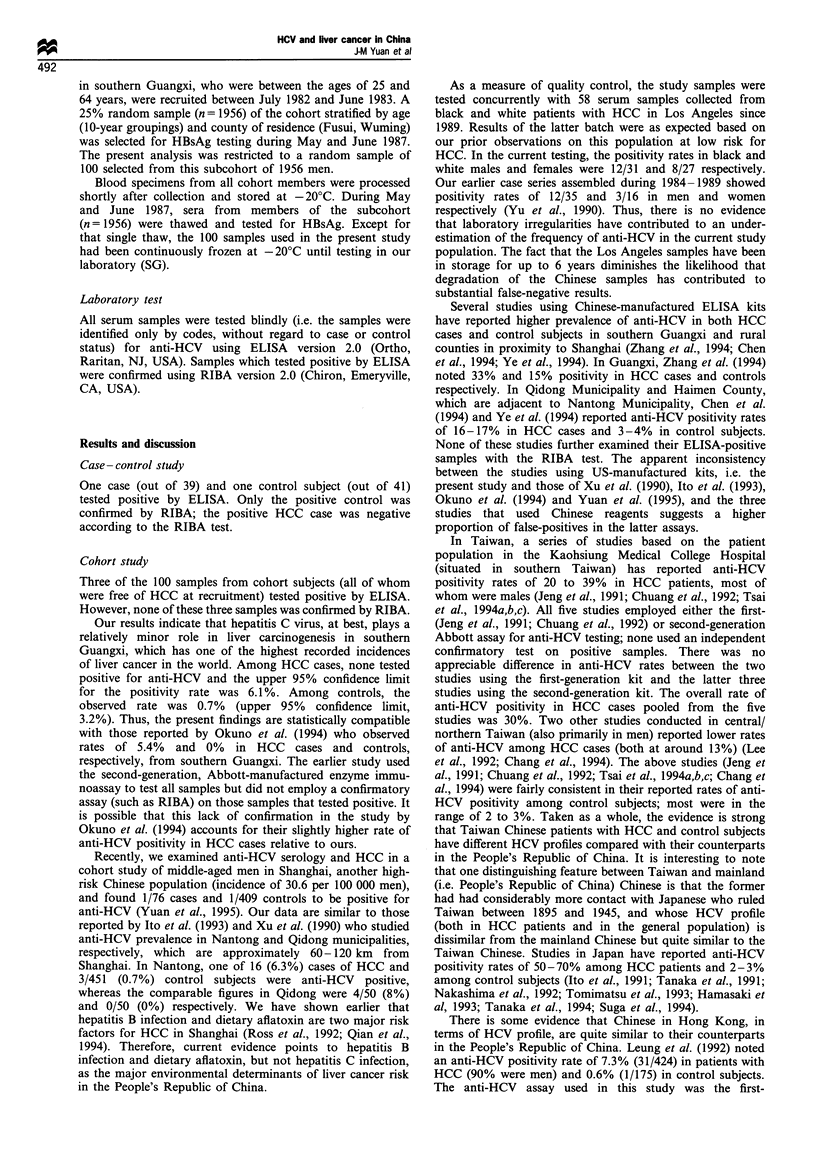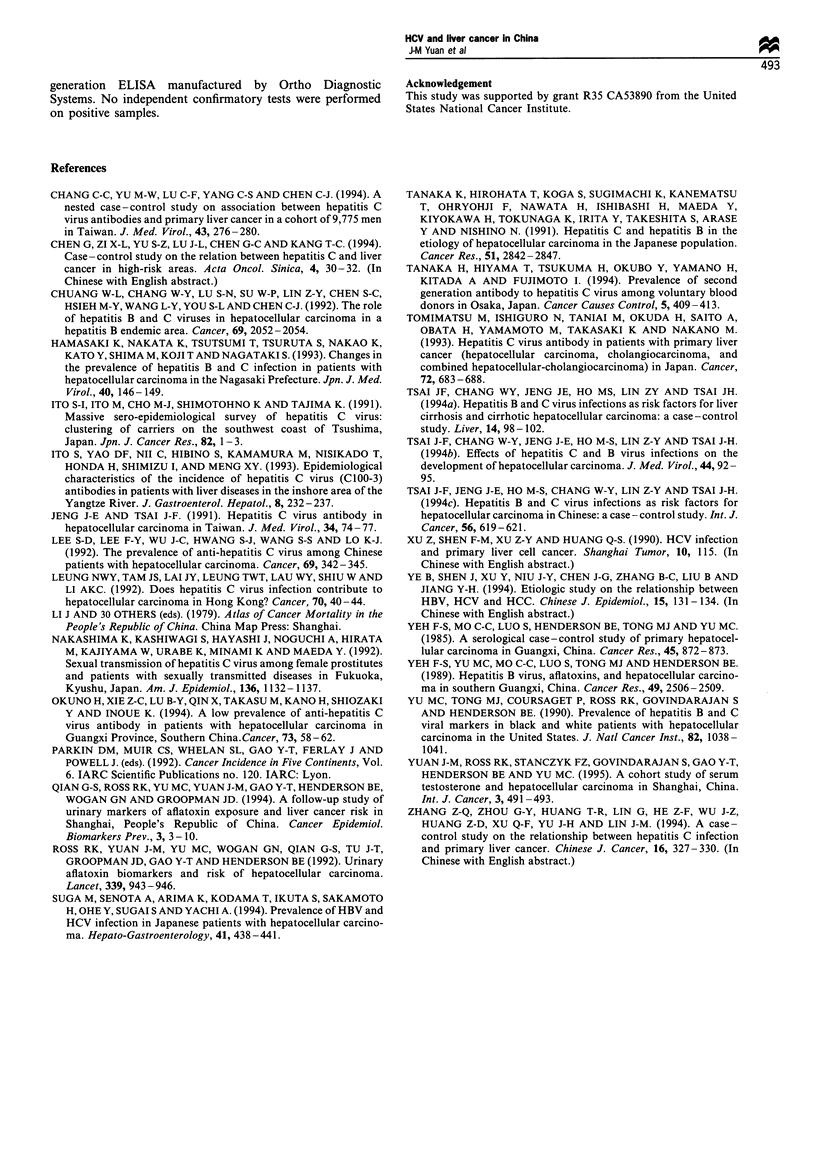# Low prevalence of hepatitis C infection in hepatocellular carcinoma (HCC) cases and population controls in Guangxi, a hyperendemic region for HCC in the People's Republic of China.

**DOI:** 10.1038/bjc.1996.389

**Published:** 1996-08

**Authors:** J. M. Yuan, S. Govindarajan, B. E. Henderson, M. C. Yu

**Affiliations:** Department of Preventive Medicine, University of Southern California School of Medicine, Los Angeles 90033, USA.

## Abstract

Southern Guangxi, China has one of the highest incidences of hepatocellular carcinoma (HCC) in the world. Serum samples collected from subjects of an earlier case-control study (39 cases, 41 controls) and from a random sampling of a residential male cohort (n = 100) were tested for antibodies for the hepatitis C virus (anti-HCV) using ELISA version 2.0 with confirmation by RIBA version 2.0. Only one of 141 (0.7%, upper 95% confidence limit, 3.2%) control subjects and none of 39 (upper 95% confidence limit, 6.07%) HCC cases tested positive for anti-HCV. Our results indicate that hepatitis C infection is not an important environmental determinant of HCC risk in this hyperendemic region.


British Journal of Cancer (1996) 74, 491-493
© 1996 Stockton Press All rights reserved 0007-0920/96 $12.00

SHORT COMMUNICATION

### Low prevalence of hepatitis C infection in hepatocellular carcinoma (HCC) cases and population controls in Guangxi, a hyperendemic region for HCC in the People's Republic of China

J-M Yuan[1], S Govindarajan[2], BE Henderson[1] and MC Yu[1]

*Department of [1]Preventive Medicine and [2]Pathology, University of Southern California School of Medicine, Los Angeles, California 90033, USA.*

**Summary** Southern Guangxi, China has one of the highest incidences of hepatocellular carcinoma (HCC) in the world. Serum samples collected from subjects of an earlier case-control study (39 cases, 41 controls) and from a random sampling of a residential male cohort ($n = 100$) were tested for antibodies for the hepatitis C virus (anti-HCV) using ELISA version 2.0 with confirmation by RIBA version 2.0. Only one of 141 (0.7%, upper 95% confidence limit, 3.2%) control subjects and none of 39 (upper 95% confidence limit, 6.07%) HCC cases tested positive for anti-HCV. Our results indicate that hepatitis C infection is not an important environmental determinant of HCC risk in this hyperendemic region.

**Keywords:** hepatitis C virus; hepatocellular carcinoma; liver cancer; China

Hepatocellular carcinoma (HCC) varies widely in incidence worldwide. It is a relatively rare malignancy in the United States and most of western Europe, but is one of the commonest cancers in large parts of Asia and sub-Saharan Africa (Parkin *et al.*, 1992). In the People's Republic of China, liver cancer represents the third and fourth commonest causes of cancer death in men and women respectively (Li *et al.*, 1979). There is considerable geographical variation in HCC incidence within China, the high-risk regions are mostly in the southeastern corner of the country with southern Guangxi exhibiting one of the highest rates of HCC incidence in the world. In Fusui County in southern Guangxi, the standardised incidence rate of liver cancer among men is 120 per 100 000 population per year, which is 50 times higher than the comparable rate in US whites (Yeh *et al.*, 1989; Parkin *et al.*, 1992).

Previously, we have shown that chronic infection with the hepatitis B (HB) virus is a primary risk factor for HCC in southern Guangxi. In a cohort study of young and middle-aged men in five rural communities in southern Guangxi, we demonstrated that approximately one-quarter of the male population are chronic HB carriers, and that liver cancer is responsible for half of all deaths in adult men. Over 90% of subsequent HCC cases were tested positive for hepatitis B surface antigen (HBsAg) at recruitment, and the incidence of HCC among HB carriers was close to 1% per year (Yeh *et al.*, 1989). There was also strong suggestive evidence that dietary aflatoxin, primarily through ingestion of mouldy corn, is an important cofactor in liver cancer development in Guangxi. We observed a 3.5-fold variation in HCC incidence between the five rural communities which constituted our cohort, although these subpopulations displayed comparable prevalence of HBsAg positivity. However, repeated dietary and food surveys have established that these communities differed in their mean exposure levels to aflatoxin B1, from a high of 51.8 mg per person per year to a low of 0.3 mg per person per year. When HCC rates in these communities were plotted against their mean load of aflatoxin B1, an almost perfectly linear relationship was noted (Yeh *et al.*, 1989).

Relatively little is known about the role of hepatitis C infection in HCC development in southern Guangxi. Okuno *et al.* (1994) tested 186 (168 men, 18 women) patients with HCC and 48 control subjects (30 men, 18 women) for antibodies to the hepatitis C virus (anti-HCV) using a second-generation, Abbott-manufactured enzyme immunoassay and noted positivity rates of 5.4% and 0% respectively. Their findings contrast with those of Zhang *et al.* (1994) who used Chinese-manufactured reagents and reported anti-HCV positivity rates of 33% and 15% in HCC cases and controls respectively. In this report, we present our findings on anti-HCV among subjects of an earlier case-control study (Yeh *et al.*, 1985) and the previously described cohort (Yeh *et al.*, 1989), both of which were conducted in southern Guangxi.

### Materials and methods

### *Case-control study*

The design of this study has been published (Yeh *et al.*, 1985). Briefly, cases were HCC patients at the clinic/ward of the Affiliated Hospital of the Guangxi Medical College in the city of Nanning. Controls were other clinic/ward patients whose present illnesses were not hepatitis or other liver diseases. Controls were individually matched to the cases by age (within 5 years), sex and clinic *vs* ward status. All subjects were recruited during 1982. The mean ages of the cases and controls were 44.3 and 44.5 years respectively. The original data set consisted of 50 cases (47 men, 3 women) and 50 controls. Owing to inadequate serum, the present analysis was restricted to 39 cases and 41 controls.

Blood specimens from all cases and controls were processed shortly after collection and stored at −20°C until analysis [for HBsAg, antibodies to the hepatitis B core antigen (anti-HBc) and antibodies to the hepatitis B surface antigen (anti-HBs)] in Los Angeles in 1983. Except for that single thaw, these specimens had been continuously frozen at −20°C until the present analysis.

### *Cohort study*

The design of this study has been published (Yeh *et al.*, 1989). Briefly, 7917 male residents of five rural communities

Correspondence: MC Yu, USC/Norris Comprehensive Cancer Center, 1441 Eastlake Avenue, Los Angeles, CA 90033-0804, USA
Received 10 November 1995; revised 4 March 1996; accepted 4 March 1996

in southern Guangxi, who were between the ages of 25 and 64 years, were recruited between July 1982 and June 1983. A 25% random sample ($n = 1956$) of the cohort stratified by age (10-year groupings) and county of residence (Fusui, Wuming) was selected for HBsAg testing during May and June 1987. The present analysis was restricted to a random sample of 100 selected from this subcohort of 1956 men.

Blood specimens from all cohort members were processed shortly after collection and stored at −20°C. During May and June 1987, sera from members of the subcohort ($n = 1956$) were thawed and tested for HBsAg. Except for that single thaw, the 100 samples used in the present study had been continuously frozen at −20°C until testing in our laboratory (SG).

*Laboratory test*

All serum samples were tested blindly (i.e. the samples were identified only by codes, without regard to case or control status) for anti-HCV using ELISA version 2.0 (Ortho, Raritan, NJ, USA). Samples which tested positive by ELISA were confirmed using RIBA version 2.0 (Chiron, Emeryville, CA, USA).

### Results and discussion

*Case–control study*

One case (out of 39) and one control subject (out of 41) tested positive by ELISA. Only the positive control was confirmed by RIBA; the positive HCC case was negative according to the RIBA test.

*Cohort study*

Three of the 100 samples from cohort subjects (all of whom were free of HCC at recruitment) tested positive by ELISA. However, none of these three samples was confirmed by RIBA.

Our results indicate that hepatitis C virus, at best, plays a relatively minor role in liver carcinogenesis in southern Guangxi, which has one of the highest recorded incidences of liver cancer in the world. Among HCC cases, none tested positive for anti-HCV and the upper 95% confidence limit for the positivity rate was 6.1%. Among controls, the observed rate was 0.7% (upper 95% confidence limit, 3.2%). Thus, the present findings are statistically compatible with those reported by Okuno *et al.* (1994) who observed rates of 5.4% and 0% in HCC cases and controls, respectively, from southern Guangxi. The earlier study used the second-generation, Abbott-manufactured enzyme immunoassay to test all samples but did not employ a confirmatory assay (such as RIBA) on those samples that tested positive. It is possible that this lack of confirmation in the study by Okuno *et al.* (1994) accounts for their slightly higher rate of anti-HCV positivity in HCC cases relative to ours.

Recently, we examined anti-HCV serology and HCC in a cohort study of middle-aged men in Shanghai, another high-risk Chinese population (incidence of 30.6 per 100 000 men), and found 1/76 cases and 1/409 controls to be positive for anti-HCV (Yuan *et al.*, 1995). Our data are similar to those reported by Ito *et al.* (1993) and Xu *et al.* (1990) who studied anti-HCV prevalence in Nantong and Qidong municipalities, respectively, which are approximately 60–120 km from Shanghai. In Nantong, one of 16 (6.3%) cases of HCC and 3/451 (0.7%) control subjects were anti-HCV positive, whereas the comparable figures in Qidong were 4/50 (8%) and 0/50 (0%) respectively. We have shown earlier that hepatitis B infection and dietary aflatoxin are two major risk factors for HCC in Shanghai (Ross *et al.*, 1992; Qian *et al.*, 1994). Therefore, current evidence points to hepatitis B infection and dietary aflatoxin, but not hepatitis C infection, as the major environmental determinants of liver cancer risk in the People's Republic of China.

As a measure of quality control, the study samples were tested concurrently with 58 serum samples collected from black and white patients with HCC in Los Angeles since 1989. Results of the latter batch were as expected based on our prior observations on this population at low risk for HCC. In the current testing, the positivity rates in black and white males and females were 12/31 and 8/27 respectively. Our earlier case series assembled during 1984–1989 showed positivity rates of 12/35 and 3/16 in men and women respectively (Yu *et al.*, 1990). Thus, there is no evidence that laboratory irregularities have contributed to an underestimation of the frequency of anti-HCV in the current study population. The fact that the Los Angeles samples have been in storage for up to 6 years diminishes the likelihood that degradation of the Chinese samples has contributed to substantial false-negative results.

Several studies using Chinese-manufactured ELISA kits have reported higher prevalence of anti-HCV in both HCC cases and control subjects in southern Guangxi and rural counties in proximity to Shanghai (Zhang *et al.*, 1994; Chen *et al.*, 1994; Ye *et al.*, 1994). In Guangxi, Zhang *et al.* (1994) noted 33% and 15% positivity in HCC cases and controls respectively. In Qidong Municipality and Haimen County, which are adjacent to Nantong Municipality, Chen *et al.* (1994) and Ye *et al.* (1994) reported anti-HCV positivity rates of 16–17% in HCC cases and 3–4% in control subjects. None of these studies further examined their ELISA-positive samples with the RIBA test. The apparent inconsistency between the studies using US-manufactured kits, i.e. the present study and those of Xu *et al.* (1990), Ito *et al.* (1993), Okuno *et al.* (1994) and Yuan *et al.* (1995), and the three studies that used Chinese reagents suggests a higher proportion of false-positives in the latter assays.

In Taiwan, a series of studies based on the patient population in the Kaohsiung Medical College Hospital (situated in southern Taiwan) has reported anti-HCV positivity rates of 20 to 39% in HCC patients, most of whom were males (Jeng *et al.*, 1991; Chuang *et al.*, 1992; Tsai *et al.*, 1994*a,b,c*). All five studies employed either the first- (Jeng *et al.*, 1991; Chuang *et al.*, 1992) or second-generation Abbott assay for anti-HCV testing; none used an independent confirmatory test on positive samples. There was no appreciable difference in anti-HCV rates between the two studies using the first-generation kit and the latter three studies using the second-generation kit. The overall rate of anti-HCV positivity in HCC cases pooled from the five studies was 30%. Two other studies conducted in central/northern Taiwan (also primarily in men) reported lower rates of anti-HCV among HCC cases (both at around 13%) (Lee *et al.*, 1992; Chang *et al.*, 1994). The above studies (Jeng *et al.*, 1991; Chuang *et al.*, 1992; Tsai *et al.*, 1994*a,b,c*; Chang *et al.*, 1994) were fairly consistent in their reported rates of anti-HCV positivity among control subjects; most were in the range of 2 to 3%. Taken as a whole, the evidence is strong that Taiwan Chinese patients with HCC and control subjects have different HCV profiles compared with their counterparts in the People's Republic of China. It is interesting to note that one distinguishing feature between Taiwan and mainland (i.e. People's Republic of China) Chinese is that the former had had considerably more contact with Japanese who ruled Taiwan between 1895 and 1945, and whose HCV profile (both in HCC patients and in the general population) is dissimilar from the mainland Chinese but quite similar to the Taiwan Chinese. Studies in Japan have reported anti-HCV positivity rates of 50–70% among HCC patients and 2–3% among control subjects (Ito *et al.*, 1991; Tanaka *et al.*, 1991; Nakashima *et al.*, 1992; Tomimatsu *et al.*, 1993; Hamasaki *et al*, 1993; Tanaka *et al.*, 1994; Suga *et al.*, 1994).

There is some evidence that Chinese in Hong Kong, in terms of HCV profile, are quite similar to their counterparts in the People's Republic of China. Leung *et al.* (1992) noted an anti-HCV positivity rate of 7.3% (31/424) in patients with HCC (90% were men) and 0.6% (1/175) in control subjects. The anti-HCV assay used in this study was the firstgeneration ELISA manufactured by Ortho Diagnostic Systems. No independent confirmatory tests were performed on positive samples.

**Acknowledgement**
This study was supported by grant R35 CA53890 from the United States National Cancer Institute.

### References

CHANG C-C, YU M-W, LU C-F, YANG C-S AND CHEN C-J. (1994). A nested case-control study on association between hepatitis C virus antibodies and primary liver cancer in a cohort of 9,775 men in Taiwan. *J. Med. Virol.*, **43**, 276-280.

CHEN G, ZI X-L, YU S-Z, LU J-L, CHEN G-C AND KANG T-C. (1994). Case-control study on the relation between hepatitis C and liver cancer in high-risk areas. *Acta Oncol. Sinica*, **4**, 30-32. (In Chinese with English abstract.)

CHUANG W-L, CHANG W-Y, LU S-N, SU W-P, LIN Z-Y, CHEN S-C, HSIEH M-Y, WANG L-Y, YOU S-L AND CHEN C-J. (1992). The role of hepatitis B and C viruses in hepatocellular carcinoma in a hepatitis B endemic area. *Cancer*, **69**, 2052-2054.

HAMASAKI K, NAKATA K, TSUTSUMI T, TSURUTA S, NAKAO K, KATO Y, SHIMA M, KOJI T AND NAGATAKI S. (1993). Changes in the prevalence of hepatitis B and C infection in patients with hepatocellular carcinoma in the Nagasaki Prefecture. *Jpn. J. Med. Virol.*, **40**, 146-149.

ITO S-I, ITO M, CHO M-J, SHIMOTOHNO K AND TAJIMA K. (1991). Massive sero-epidemiological survey of hepatitis C virus: clustering of carriers on the southwest coast of Tsushima, Japan. *Jpn. J. Cancer Res.*, **82**, 1-3.

ITO S, YAO DF, NII C, HIBINO S, KAMAMURA M, NISIKADO T, HONDA H, SHIMIZU I, AND MENG XY. (1993). Epidemiological characteristics of the incidence of hepatitis C virus (C100-3) antibodies in patients with liver diseases in the inshore area of the Yangtze River. *J. Gastroenterol. Hepatol.*, **8**, 232-237.

JENG J-E AND TSAI J-F. (1991). Hepatitis C virus antibody in hepatocellular carcinoma in Taiwan. *J. Med. Virol.*, **34**, 74-77.

LEE S-D, LEE F-Y, WU J-C, HWANG S-J, WANG S-S AND LO K-J. (1992). The prevalence of anti-hepatitis C virus among Chinese patients with hepatocellular carcinoma. *Cancer*, **69**, 342-345.

LEUNG NWY, TAM JS, LAI JY, LEUNG TWT, LAU WY, SHIU W AND LI AKC. (1992). Does hepatitis C virus infection contribute to hepatocellular carcinoma in Hong Kong? *Cancer*, **70**, 40-44.

LI J AND 30 OTHERS (eds). (1979). *Atlas of Cancer Mortality in the People's Republic of China*. China Map Press: Shanghai.

NAKASHIMA K, KASHIWAGI S, HAYASHI J, NOGUCHI A, HIRATA M, KAJIYAMA W, URABE K, MINAMI K AND MAEDA Y. (1992). Sexual transmission of hepatitis C virus among female prostitutes and patients with sexually transmitted diseases in Fukuoka, Kyushu, Japan. *Am. J. Epidemiol.*, **136**, 1132-1137.

OKUNO H, XIE Z-C, LU B-Y, QIN X, TAKASU M, KANO H, SHIOZAKI Y AND INOUE K. (1994). A low prevalence of anti-hepatitis C virus antibody in patients with hepatocellular carcinoma in Guangxi Province, Southern China.*Cancer*, **73**, 58-62.

PARKIN DM, MUIR CS, WHELAN SL, GAO Y-T, FERLAY J AND POWELL J. (eds). (1992). *Cancer Incidence in Five Continents*, Vol. 6. IARC Scientific Publications no. 120. IARC: Lyon.

QIAN G-S, ROSS RK, YU MC, YUAN J-M, GAO Y-T, HENDERSON BE, WOGAN GN AND GROOPMAN JD. (1994). A follow-up study of urinary markers of aflatoxin exposure and liver cancer risk in Shanghai, People's Republic of China. *Cancer Epidemiol. Biomarkers Prev.*, **3**, 3-10.

ROSS RK, YUAN J-M, YU MC, WOGAN GN, QIAN G-S, TU J-T, GROOPMAN JD, GAO Y-T AND HENDERSON BE (1992). Urinary aflatoxin biomarkers and risk of hepatocellular carcinoma. *Lancet*, **339**, 943-946.

SUGA M, SENOTA A, ARIMA K, KODAMA T, IKUTA S, SAKAMOTO H, OHE Y, SUGAI S AND YACHI A. (1994). Prevalence of HBV and HCV infection in Japanese patients with hepatocellular carcinoma. *Hepato-Gastroenterology*, **41**, 438-441.

TANAKA K, HIROHATA T, KOGA S, SUGIMACHI K, KANEMATSU T, OHRYOHJI F, NAWATA H, ISHIBASHI H, MAEDA Y, KIYOKAWA H, TOKUNAGA K, IRITA Y, TAKESHITA S, ARASE Y AND NISHINO N. (1991). Hepatitis C and hepatitis B in the etiology of hepatocellular carcinoma in the Japanese population. *Cancer Res.*, **51**, 2842-2847.

TANAKA H, HIYAMA T, TSUKUMA H, OKUBO Y, YAMANO H, KITADA A AND FUJIMOTO I. (1994). Prevalence of second generation antibody to hepatitis C virus among voluntary blood donors in Osaka, Japan. *Cancer Causes Control*, **5**, 409-413.

TOMIMATSU M, ISHIGURO N, TANIAI M, OKUDA H, SAITO A, OBATA H, YAMAMOTO M, TAKASAKI K AND NAKANO M. (1993). Hepatitis C virus antibody in patients with primary liver cancer (hepatocellular carcinoma, cholangiocarcinoma, and combined hepatocellular-cholangiocarcinoma) in Japan. *Cancer*, **72**, 683-688.

TSAI JF, CHANG WY, JENG JE, HO MS, LIN ZY AND TSAI JH. (1994*a*). Hepatitis B and C virus infections as risk factors for liver cirrhosis and cirrhotic hepatocellular carcinoma: a case-control study. *Liver*, **14**, 98-102.

TSAI J-F, CHANG W-Y, JENG J-E, HO M-S, LIN Z-Y AND TSAI J-H. (1994*b*). Effects of hepatitis C and B virus infections on the development of hepatocellular carcinoma. *J. Med. Virol.*, **44**, 92-95.

TSAI J-F, JENG J-E, HO M-S, CHANG W-Y, LIN Z-Y AND TSAI J-H. (1994*c*). Hepatitis B and C virus infections as risk factors for hepatocellular carcinoma in Chinese: a case-control study. *Int. J. Cancer*, **56**, 619-621.

XU Z, SHEN F-M, XU Z-Y AND HUANG Q-S. (1990). HCV infection and primary liver cell cancer. *Shanghai Tumor*, **10**, 115. (In Chinese with English abstract.)

YE B, SHEN J, XU Y, NIU J-Y, CHEN J-G, ZHANG B-C, LIU B AND JIANG Y-H. (1994). Etiologic study on the relationship between HBV, HCV and HCC. *Chinese J. Epidemiol.*, **15**, 131-134. (In Chinese with English abstract.)

YEH F-S, MO C-C, LUO S, HENDERSON BE, TONG MJ AND YU MC. (1985). A serological case-control study of primary hepatocellular carcinoma in Guangxi, China. *Cancer Res.*, **45**, 872-873.

YEH F-S, YU MC, MO C-C, LUO S, TONG MJ AND HENDERSON BE. (1989). Hepatitis B virus, aflatoxins, and hepatocellular carcinoma in southern Guangxi, China. *Cancer Res.*, **49**, 2506-2509.

YU MC, TONG MJ, COURSAGET P, ROSS RK, GOVINDARAJAN S AND HENDERSON BE. (1990). Prevalence of hepatitis B and C viral markers in black and white patients with hepatocellular carcinoma in the United States. *J. Natl Cancer Inst.*, **82**, 1038-1041.

YUAN J-M, ROSS RK, STANCZYK FZ, GOVINDARAJAN S, GAO Y-T, HENDERSON BE AND YU MC. (1995). A cohort study of serum testosterone and hepatocellular carcinoma in Shanghai, China. *Int. J. Cancer*, **3**, 491-493.

ZHANG Z-Q, ZHOU G-Y, HUANG T-R, LIN G, HE Z-F, WU J-Z, HUANG Z-D, XU Q-F, YU J-H AND LIN J-M. (1994). A case-control study on the relationship between hepatitis C infection and primary liver cancer. *Chinese J. Cancer*, **16**, 327-330. (In Chinese with English abstract.)